# Public patient views of artificial intelligence in healthcare: A nominal group technique study

**DOI:** 10.1177/20552076211063682

**Published:** 2021-12-15

**Authors:** Omar Musbahi, Labib Syed, Peter Le Feuvre, Justin Cobb, Gareth Jones

**Affiliations:** 1 MSK Lab, 4615 Imperial College London, Charing Cross Campus, Hammersmith, London, UK

**Keywords:** Artificial intelligence, Digital health, Patient, Qualitative

## Abstract

**Objectives:**

The beliefs of laypeople and medical professionals often diverge with regards to disease, and technology has had a positive impact on how research is conducted. Surprisingly, given the expanding worldwide funding and research into Artificial Intelligence (AI) applications in healthcare, there is a paucity of research exploring the public patient perspective on this technology. Our study sets out to address this knowledge gap, by applying the Nominal Group Technique (NGT) to explore patient public views on AI.

**Methods:**

A Nominal Group Technique (NGT) was used involving four study groups with seven participants in each group. This started with a silent generation of ideas regarding the benefits and concerns of AI in Healthcare. Then a group discussion and round-robin process were conducted until no new ideas were generated. Participants ranked their top five benefits and top five concerns regarding the use of AI in healthcare. A final group consensus was reached.

**Results:**

Twenty-Eight participants were recruited with the mean age of 47 years. The top five benefits were: Faster health services, Greater accuracy in management, AI systems available 24/7, reducing workforce burden, and equality in healthcare decision making. The top five concerns were: Data cybersecurity, bias and quality of AI data, less human interaction, algorithm errors and responsibility, and limitation in technology.

**Conclusion:**

This is the first formal qualitative study exploring patient public views on the use of AI in healthcare, and highlights that there is a clear understanding of the potential benefits delivered by this technology. Greater patient public group involvement, and a strong regulatory framework is recommended.

## Introduction

Understanding that the beliefs of laypeople and medical professionals often diverge with regards to disease and technology has had a positive impact on how research is conducted^1, 2^. This has led to close patient involvement at the development stage of a research or technology proposal, to ensure that the work is relevant and useful^2^.

Recent studies demonstrating that AI can be more accurate than even experienced clinicians in diagnosing conditions such as breast cancer, retinal disease, and skin cancer^3–5^, has led to calls for its rapid integration into healthcare delivery ^6, 7^. However, for this to be successful, it is essential to understand the public patient perspective, so that any concerns can be addressed at the outset.

Surprisingly, given the expanding worldwide funding and research into AI applications in healthcare^8^, there is a paucity of research exploring the public patient perspective on this technology^9, 10^. These studies have also typically been limited to questionnaires with a focus on radiological AI applications. Our study sets out to address this knowledge gap, by applying the Nominal Group Technique (NGT) to explore patient public views on AI, with the specific aim of establishing a consensus on the perceived five most important potential benefits and risks of AI in healthcare.

## Methods

Local institutional ethics approval was obtained for this study (ICREC 20IC6017), and consent obtained from all participants. A Nominal Group Technique (NGT) was used for each session. NGT is a validated focused group interview that promotes the generation of ideas and issues pertaining to the topic in question^11^. It is a powerful qualitative development technique to analyse healthcare issues and has also been employed to identify priorities in healthcare^11^.

A target of four study groups with seven participants in each group was set based on the published recommendations of the NGT as well as previously published studies^12–14^. Recruitment was via both a university patient involvement mailing list and through a nationwide patient public initiative platform. The university patient public involvement mailing list consists of over 500 registered emails of public members from around the North West London area. The nationwide patient public initiative platform has over 1000 registered public members from around the United Kingdom(UK) and is commonly used by researchers around the UK for patient public involvement^15^. We received 51 respondents interested in taking part in the study. From these, we excluded anyone with a background in healthcare or computer science/artificial intelligence, anyone not fluent in English, and applicants under the age of 18. Forty patients were then categorised by age into two groups (>50 and <50 years old). This purposive age sampling was to ensure that there was a range of different demographics in each focus group as age is considered one of the biggest determinants of digital technology use^16–18^. Fourteen participants were then randomly selected from each group to take part. These were then placed into 4 focus groups consisting of 7 participants each.

A questionnaire was emailed to participants before the group session to establish their baseline knowledge and views regarding artificial intelligence Appendix 1. Each group session was facilitated by two of the authors (OM and LS). To ensure all participants understood the subject matter sufficiently to engage in the subsequent discussions, at the start of each session, a short pre-recorded objective presentation was played, describing in plain English the following clinical studies of AI; Rapid Triage for COVID-19^19^, Breast Cancer Screening ^5^, skin cancer^20^ and retinal pathology^21^.

**Figure 1.**
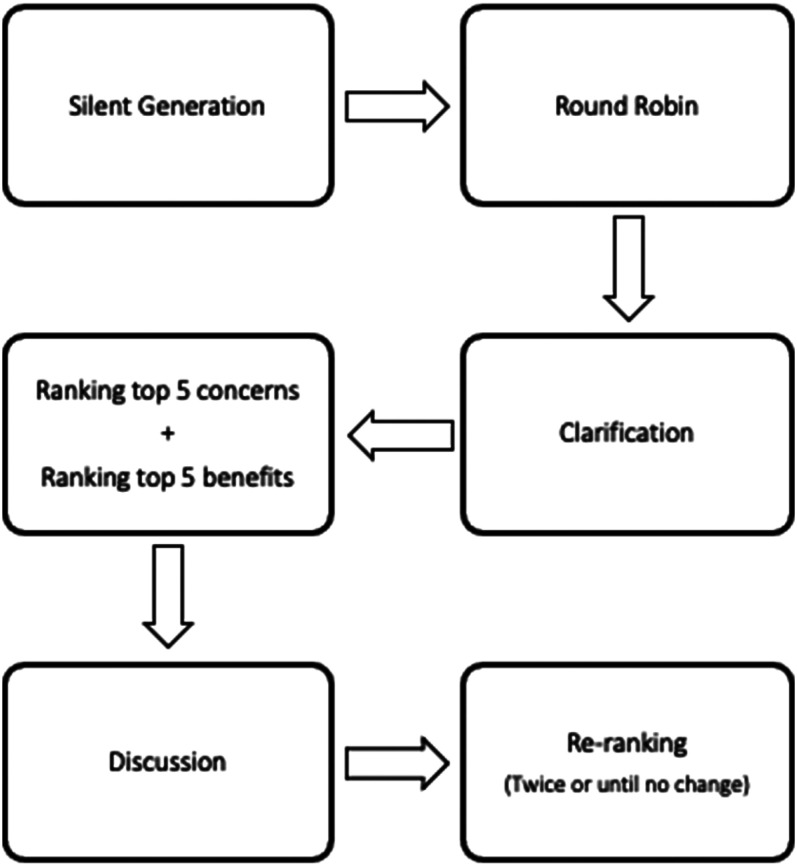
Nominal Group Technique flowchart in each focus group

Each focus group followed a standard NGT cycle^12^ (Figure 1): this started with a silent generation of ideas to allow individuals to develop their own thoughts regarding the benefits and concerns of Artificial Intelligence in Healthcare. This was followed by a group discussion where each participant listed one of their ideas in turn. These ideas were written down for all participants to see, and the round-robin process continued until no new ideas were generated. Participants were then asked to rank what they perceived as the top five benefits and top five concerns regarding the use of artificial intelligence in healthcare, and each participant's ranking was discussed within the group. Participants were then allowed to re-rank their top five benefits and top five concerns. A tally of these results was used to determine the overall final rankings for each group. Two members of the research team (OM and LS) then combined the tallies for all 4 groups to produce the consensus between all 4 groups for the top 5 benefits and concerns of artificial intelligence in healthcare.

Each focus group was also recorded and subsequently anonymously transcribed. A summative content analysis technique was performed by two members of the research team(OM and LS) ^22^. This was used to provide further information on key themes discussed in the focus groups.

## Results

### Recruitment

Twenty-Eight participants (four focus groups, with seven participants each) were recruited (Table 1). Sixty-one per cent were female, and the mean age was 47 years. Sixty-nine per cent of participants were Caucasian, 8% were mixed race, and 23% of participants were of Indian subcontinent Asian origin.

**Table 1.** Characteristics of Nominal Groups

	Group 1 (n=7)	Group 2 (n=7)	Group 3(n=7)	Group 4(n=7)
Participant age(mean, range)	46(23-67)	54(35-59)	31(23-79)	57(25-78)
Gender ratio (Female: Male)	7:0	5:2	2:5	3:4

### Questionnaire

**Figure 2.**
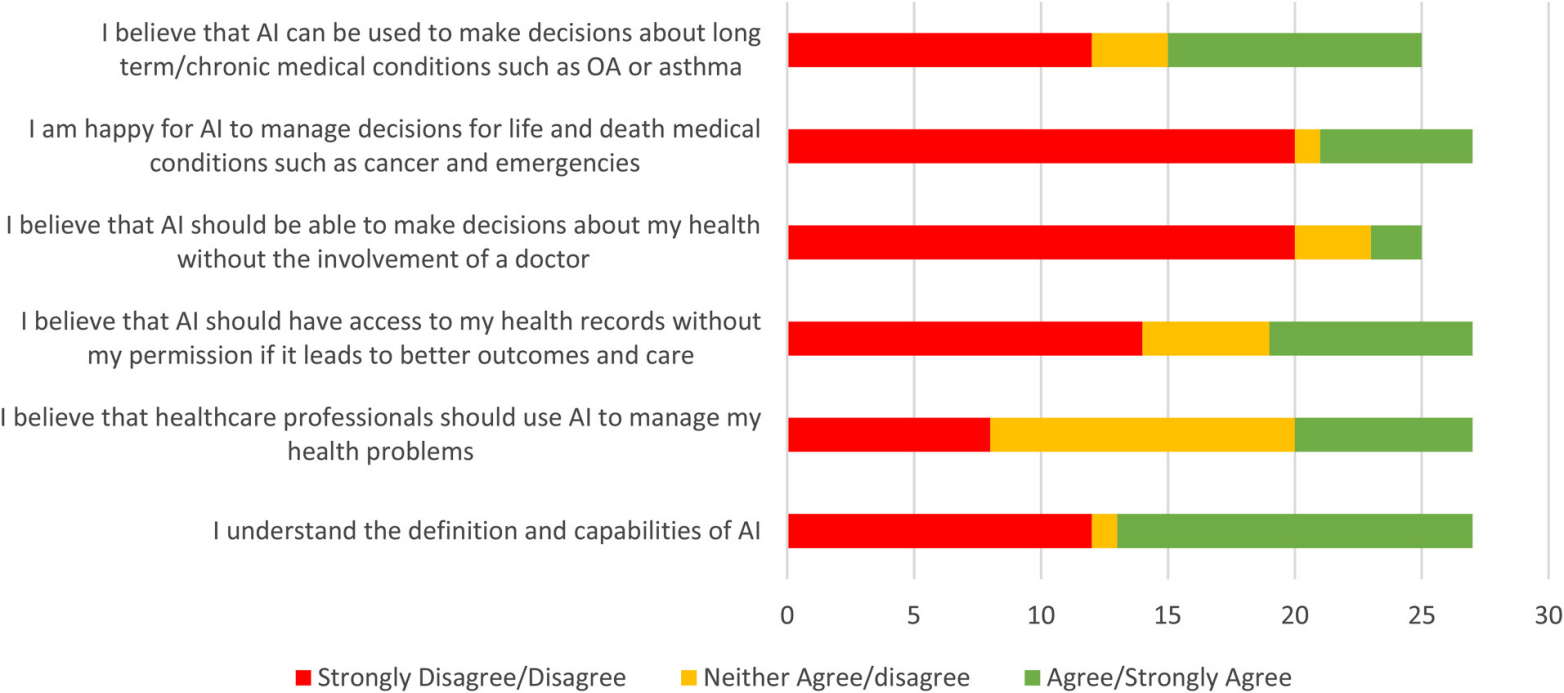
Graph showing the results of the pre-focus group questionnaire

Twenty-seven participants (96%) completed the initial questionnaire (Figure 2). Approximately half (52%) of the participants felt that they understood the definitions and capabilities of AI. Eighty per cent felt that the AI should not be used to manage health without the involvement of a doctor.

### Nominal group technique

In total during the silent generation stage, thirteen benefits and fifteen concerns regarding the use of AI were identified by the focus groups (Table 2). Some clarification by the facilitators was required at this stage regarding the definition of AI, with 3 participants initially confusing AI with robotics.

**Table 2.** NGT showing the main ideas generated in the four NGT sessions

Summary of ideas generated by the four NGT focus groups
Benefits of Artificial Intelligence in Healthcare Faster and quicker diagnosis reached by an AI system Artificial intelligence algorithms can use the data to spot trends and patterns that humans are unable to determine The capability of more advanced predictions in health outcomes The ability of AI to improve and learn from mistakes AI potential as a triage service Available 24/7 and more efficient system AI will always have consistence Reduces admin tasks to let doctors do their jobs Artificial intelligence can be used as a support tool to provide more information Artificial Intelligence has the potential to provide equal healthcare access to everyone Has the potential for saving costs in healthcare services The potential use of AI in health research
*Concerns of Artificial Intelligence in Healthcare* Hacking and cybersecurity of health data Issue of privacy and where any health information artificial intelligence systems are kept Concern about the real accuracy of AI in diagnosing AI requires a high amount of good data which may not be there The use of AI may result in significant job losses Who is responsible if AI produces a bad health outcome? Concern about the use of poor data and misinformation Losing the emotional side of patient-doctor relationships No guidelines or framework to monitor the creation of artificial intelligence algorithms The role of AI in end-of-life care Use of AI created in rich countries to dictate health services in poorer countries Companies with hidden agendas selling their AI algorithms May cause certain professions such as radiologists to deskill or affect learning Limited use in using Artificial Intelligence in mental health Using the AI algorithms when they’re not ready or complete

**Figure 3.**
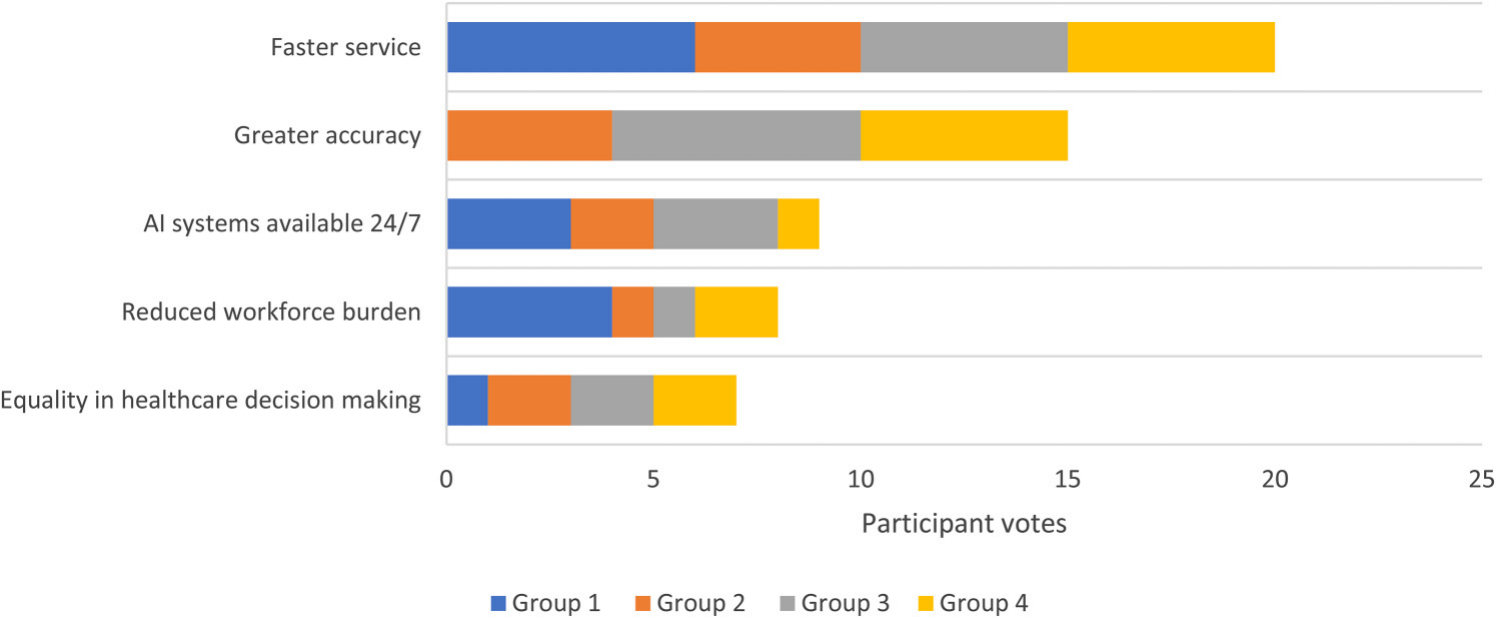
The top 5 benefits of AI in healthcare as determined by the number of votes across all groups

**Figure 4.**
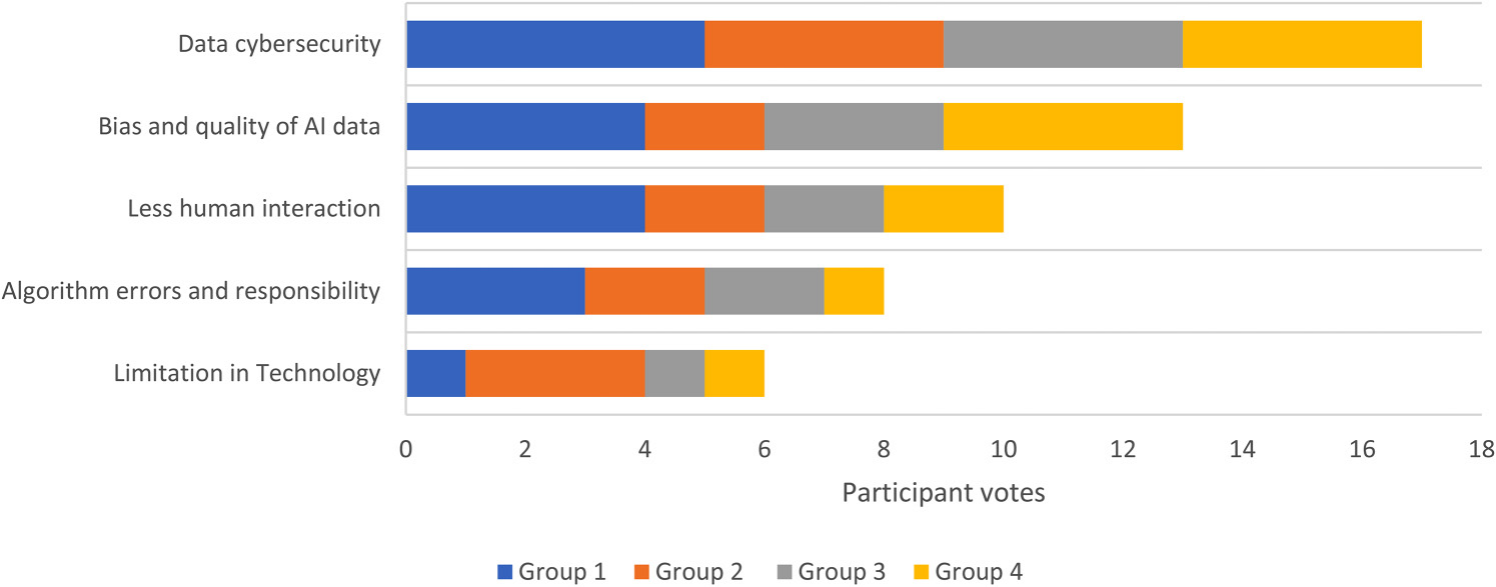
The top 5 concerns of AI in healthcare as determined by the number of votes across all groups

For the final ranking process, there was an average of two cycles per group. Table 3 details these rankings for each group. Taking the groups together, the overall top five benefits were: (1) Faster health services (2) Greater accuracy in management (3) AI systems available 24/7 (4) Reducing workforce burden (5) Equality in healthcare decision making (Figure 3). The top five concerns were: (1) Data cybersecurity (2) Bias and quality of AI data (3) Less human interaction (4) Algorithm errors and responsibility (5) Limitation in technology (Figure 4).

**Table 3.** The final Top 5 concerns and benefits of the NGT focus group sessions.

	Group 1–2 ranking cycles	Group 2–2 ranking cycles	Group 3–2 ranking cycles	Group 4–3 ranking cycles
Top 5 Benefits of AI in healthcare	The belief that AI will be more accurate in health diagnosis and management AI will lead to faster diagnosis of disease AI will have a beneficial role in personalised medicine AI algorithms will lead to greater efficiency in primary and secondary care AI systems are available 24/7	The speed and efficiency of decision making AI systems are available 24/7 AI can reduce the burden on healthcare AI will have more consistency in health decisions The potential use of AI in spotting trends in disease progression	AI will reduce the errors AI will ensure there is a lower burden on staff AI will be faster and more efficient Potential role in primary care and triage Financial savings	AI will be more efficient and quicker in deciding the best management AI will lead to faster diagnosis by analysing greater data AI systems available 24/7 The potential use of AI in disease prediction AI systems will lead to equal health decisions
Top 5 concerns of AI in healthcare	Who will be responsible for the AI algorithms? The loss of human interaction What will be the consequences of errors and misinformation generated by AI systems? Regulation of artificial intelligence in the Health Service Issues of data security and cybersecurity	Errors in AI systems Concern regarding the quality of data in the AI algorithms Loss of human interaction AI not able to identify grey areas to make decisions The application of AI as a decision aid in different cultures and settings	Data security and storage Loss of human interaction if reliance on AI Who is responsible if things go wrong with an AI system? Cost and IT maintenance of AI in Health service Software companies with hidden agendas using health data	Loss of Human interaction Data security Consent of data use Errors in AI systems and spotting them AI not relevant or applicable to all aspects of healthcare

#### Content analysis

Transcript analysis of the focus groups highlighted three overarching themes: automation of healthcare decision making, the use of AI as a decision aid, and health data security.

#### Automation of healthcare decision making

All groups reached a unifying consensus that the automation of healthcare decision making is a positive step forward. Many participants felt that there was an existing strain on the healthcare system and any form of digital advancement to ease this pressure would be positive. One participant described this:

‘*Using AI can just, reduce the burden on the health workforce, meaning doctors can do what they’re supposed to do”.(Group 1, participant 4)*

However, there was concern regarding the quality and homogeneity of the data used in the algorithms:

“*The data used to create the Algorithm may not represent the vast majority of patients, to me, this makes artificial intelligence dangerous, the data just may not be there”(Group 2, participant 5)*.

#### Artificial intelligence as a decision aid

There was a consensus that AI should be used as a support tool rather than a primary healthcare decision-maker for patients. Indeed, one participant felt that this will be its primary role for the foreseeable future:

‘*Whilst AI can be very good at predicting what will happen, the AI should only be used as a decision aid rather than a decision-maker and I don't think that's going to change for a long time to come. The technology just isn't there yet” (Group 2, participant 4)*

From the group discussion, 17 participants were concerned that reliance on artificial intelligence may impact medical workforce training and have a negative impact on the skillset of the health workforce.

#### Health data security

Data security was the most common concern regarding the use of AI, with numerous discussions on this issue. All four focus groups reached a unanimous agreement that there should be a regulatory framework for the use of AI when handling NHS data. Four participants felt that the government, and not the health service, have a responsibility to ensure that there is a regulatory process in AI health data security. As one group member summarised:

“*We need to be aware that we know nothing about who these people that are creating these AI algorithms, they can be anyone and they’d have access to all our data” (Group 1, participant 2)*.

This also appeared to invoke a response in NGT focus group 3. Over 50% of participants mentioned their concerns:

“*Who is responsible if an AI algorithm makes a mistake” (Group 1&2, participants 3&4 respectively)*.

## Discussion

All the participants could see the potential benefit of using AI in the healthcare sector: (1) Faster service, (2) Greater accuracy, (3) AI systems available 24/7, (4) Reduced workforce burden, (5) Equality in healthcare decision making. However, participants also identified concerns about its use (1) Data cybersecurity, (2) Bias and quality of AI data, (3) Less human interaction, (4) Algorithm errors and responsibility, (5) Limitation in Technology. These points all sit within three common themes: automation of healthcare data, data security and artificial intelligence as a decision aid.

This study is the first to use a validated qualitative methodology such as the Nominal Group Technique to assess patient/public perception of AI, with few comparative studies. York et al found that there was high confidence from patients in the role of AI assistance in interpreting skeletal radiology (7/10), but they remained significantly more confident in their clinician's ability to correctly interpret the imaging (9/10)^9^. The participants were also significantly more confident in AI as a decision aid for clinicians rather than as a standalone treatment tool, which is consistent with our findings that patients are concerned about both the accuracy of AI, the equality of its treatment decisions, and the loss of human interaction. A survey of US primary care providers’ attitudes identified a similar theme with 76% of providers accepting AI in a triage role and only 24% were against AI autonomy ^23^. This is further supported by a systematic review of healthcare chatbots, which often use AI algorithms, and generated mixed reviews for qualitative user perception with users disliking the lack of personal interaction with chatbots ^24^. However, consistent with the benefits of AI identified in our study, others felt that AI chatbots were a significant aid to physicians and healthcare cost reductions.

For AI to function optimally, there is a need for large, multilevel, integrated data sets, which are likely to increase in size and complexity as this technology plays an increasing role in healthcare^25^. However, we currently only use a fraction of the available data for health care informatics^26^. Naturally, this requirement for shared big data sets generates concern regarding health data privacy, as identified in our study as the participants’ main concern with AI. This has also been highlighted recently by the mainstream media with widespread concern and distrust regarding the National Health Service(NHS) Digital's plans to centralise anonymised patient data^27^. Trust in technology is vital because the information it provides might have life and death implications^28^. A significant proportion of all the focus group participants in our study felt that there should be a regulatory framework. On April 2^nd^ 2019, the FDA published a landmark guideline entitled ‘Proposed Regulatory Framework for modifications to Artificial Intelligence/Machine Learning(AI/ML)’ to address the issue of monitoring self-learning algorithms^29^. This proposed that Artificial Intelligence should be identified separately to standard “Software as Medical Device(SaMD)”^29^. As AI algorithms have the unique ability to learn from real-world feedback and improve their performance, they are unique. The FDA has since identified a separate framework for AI-SaMD. AI creators submit a marketing application to the FDA before the initial distribution of their medical device, with the submission type and data requirements based on the risk of the AI-SaMD notification or premarket approval application. In the UK, the MHRA (Medicines Health Regulation Authority) has issued a less AI-specific set of regulations for software algorithms, detailing the necessity of a CE mark and post-market surveillance^30, 31^. Furthermore, in July 2020, the UK information Commissioners Office (ICO) published guidance on AI and Data Privacy^32^. This guidance sets out a framework for auditing AI systems for compliance with data protection obligations under the General Data Protection Regulation (GDPR) and the UK Data Protection Act 2018^32^. The aim of this is to ensure good data practice in AI.

Bias and quality of AI data was the second most identified area of concern by the NGT. Deep learning algorithms are entirely dependent on the data used for training, and it is recognised that algorithms derived from homogenous population data might exacerbate racial and other disparities in healthcare^33^. This has been well described in several studies and a literature review of 52 papers using natural language processing (NLP) models in mental health found that no model addressed the possible biases in their development^34^. Another example is ImageNet, which is the most widely used data set for Deep Neural Network applications, but 45% of its data comes from the USA with less than 10% from developing counties ^35^, a lack of geodiversity which lends itself to racial and societal bias. However, if concerns regarding bias can be addressed through reporting of algorithmic performance for diverse ethnic, racial, age and gender groups ^33^, our NGT identified the public recognition that AI has the potential to improve healthcare equality by delivering high quality decision making irrespective of clinician expertise. The recent World Health Organisation(WHO) guidance on Ethics & Governance of Artificial Intelligence for Health is a major step forward in recognising the importance of ethics and human rights at the centre of Artificial Intelligence^36^. This sets six principles to limit the risks to AI for health. They detail the importance of designing AI systems to reflect the diversity of socio-economic and health-care settings alongside digital skills training. The other key principles are to protect human autonomy, safeguarding privacy, inclusivity, ensure safety and accuracy and promoting AI that is responsive and sustainable^36^. These principles corroborate our NGT study findings.

### Future research

The findings of the study promote the need to explore further the human-computer interface and how human variance and psychosocial need can be accommodated into AI algorithms. A key area of further research as identified in the NGT, is methods to limit the bias decision making to reflect the diverse socio-economic populations. This invariably requires greater quality and diverse data. Perhaps there should be greater validation and testing of AI datasets on different international data. Furthermore, this study did not explore different cultural and racial views on the adoption of AI in healthcare. This is an area for further exploration to improve implementation of AI as previous studies have identified socio-ethnic different views in digital health and technology^37, 38^.

### Limitations

Whilst the NGT is an established method of generating ideas regarding a topic, it does have limitations. It is limited to a ‘single topic meeting’ and hence arguably the depth of participant understanding of a topic cannot be fully explored^39^. Furthermore, Steward et al have demonstrated that the rigidity and formality of the process may be a limiting factor in developing a true consensus on a topic^40^. Ours was a small-scale study; however, the Nominal Group Technique is validated to be used at a theoretical level for a general application. The UK Healthcare research system advocates the use of Public Patient Initiatives in prioritising healthcare research, commissions, and services. Although there is no recommended methodology for these purposes^41^, the NGT is the most validated method of assessing patient public perspective on health-related interventions using small groups.

## Conclusion

This is the first formal qualitative study exploring patient public views on the use of AI in healthcare, and highlights that there is a clear understanding of the potential benefits delivered by this technology. However, to maintain public trust in AI to improve healthcare, the concerns identified in this study need to be addressed. Greater patient public group involvement, and a strong regulatory framework is recommended.

## Appendix

Appendix  1. Questionnaire to assess patient baseline understanding and views on AI

Appendix 1. (continued)
